# Have gender and ethnic disparities in ophthalmology disappeared? Insights from a workforce-based study in Israel (2006–2021)

**DOI:** 10.1186/s13584-024-00664-2

**Published:** 2025-01-10

**Authors:** Asaf Israeli, Eytan Z. Blumenthal, Achia Nemet, Shiri Zayit-Soudry, Hadas Pizem, Eedy Mezer

**Affiliations:** 1 Ruth and Bruce Rappaport Faculty of Medicine, Technion-Israel Institute of Technology, POB 9907, Haifa, Israel; 2 Department of Ophthalmology, Rambam Health Care Campus, Haifa, Israel; 3 Department of Ophthalmology, Assuta Ashdod Medical Center, Ashdod, Israel

**Keywords:** Ethnicity, Gender, Ophthalmologists, Residents, Trends

## Abstract

**Background:**

Workforce diversity in healthcare has been shown to improve the quality of patient care. A paucity of data exists globally on this subject in ophthalmology. The purpose of this study was to analyze nationwide trends in gender-, ethnic- and country of graduation disparities among ophthalmologists in Israel.

**Methods:**

Cross-sectional, workforce-based study using data retrieved from the Israeli Ministry of Health. Data included gender, ethnicity, and medical graduation country. Proportions and trends among new residents and board-certified ophthalmologists (BCO) were assessed.

**Results:**

During 2006–2021, 18,624 medical licenses were issued (41.7% Israeli Medical Graduates (IsrMGs), 42.2% female), average yearly increase (AYI) was 6.9%, females and IsrMGs had an average yearly decrease of 1% and 0.53%, respectively. 561 physicians began ophthalmology residency (57.5% male, 75% Jewish, 69.9% IsrMGs), reflecting a 6.2% total AYI, but 8.1% and 4.8% for female versus male residents, respectively. There were fewer female residents despite population and graduate pool adjustments (*p* = 0.002 and *p* = 0.002, respectively), but differences disappeared after 2015 (*p* = 0.52). Arab and Jewish residents AYIs were 6.4% and 5.7% respectively, with ethnic differences elucidated by adjusting for population sizes (*p* = 0.097). BCO densities in 2006 and 2021 were 7.5 and 9.06, respectively, with a 1.3% AYI (*p* < 0.001). Proportions of female and Arab BCO were lower than expected based on population proportions. (*p* < 0.001 and *p* < 0.001, respectively). Gender-differences remained after adjusting for population sizes (*p* < 0.001), but AYIs for female and male BCO were 1.38% and 1.15%, respectively. AYIs for Arab and Jewish BCO were 12% and 0.61%, respectively.

**Conclusions:**

Jewish and male dominance was seen among Israeli BCOs and was unrelated to population size or graduate distribution. Among new ophthalmology residents, Arab representation was adequate to their population proportion. In early years, male predominance was noted, however this disparity was no longer evident after 2015. These trends are encouraging, and efforts should be made to ensure the field remains inclusive and representative of the broader population.

**Supplementary Information:**

The online version contains supplementary material available at 10.1186/s13584-024-00664-2.

## Introduction

Disparity in the ophthalmology field is a widely studied subject, encompassing both health-related issues and social aspects such as education, work opportunities, and networking. Previous studies have demonstrated numerous health and work inequalities regarding gender, ethnicity, and even medical graduation status in obtaining residency positions, surgical experience during residency, as well as various aspects of research (e.g., authorship, review authorships, citations, etc.) [1–8].

A paucity of data regarding ophthalmology-related disparities and their evolving patterns among the medical workforce exists with the exception of the US and the UK. Women and ethnic minorities in the US, for example, are known to be underrepresented in the medical field and ophthalmology specifically. These disparities exist despite the fact that workplace diversity, a sought-after goal, has been proved to improve clinical outcomes and patient satisfaction [9–11]. In the UK, a staggering 74% of ophthalmologist consultants are male, and the number of black ophthalmologists, for example, is scant [12]. Such information, albeit important, has yet to be studied in Israel from a reliable comprehensive database—and was mainly studied using small sample-size surveys [13]. Israel maintains a nationwide registry of physicians under the Health Workforce and Infrastructure Forecast (HWIF) division in the Israeli Ministry of Health (MOH)—which can be utilized for this purpose.

Affirmative action policies have been implemented in four Israeli flagship universities during the study period, but not in residency applications [14]. This workforce-based study investigates disparities in Israel on a nationwide level, by evaluating long-term trends among medical graduates, new ophthalmology residents (OR), and board-certified ophthalmologists (BCO) and analyzes the effects of these policies. This study assesses the current situation among various demographic and educational subgroups in order to help establish a plan to bridge the gaps, striving for better representation and to minimize systemic inequalities.

## Materials and methods

### Ethical issues

This cross-sectional workforce-based study and data collection conform to all local regulations and adhere to the tenets of the Declaration of Helsinki. Approval was obtained from the Institutional Review Board (IRB) of Rambam Health Care Campus, Haifa, Israel (No. 0008-23-RMB-D), consistent with standard institutional policy.

### Data collection

Data were retrieved from the financial and strategic planning administration at the Israeli MOH, which develops and oversees strategic planning with regard to workforce in the medical field, in order to inform the ministry’s policy. All licensed physicians in Israel holding a medical doctor (MD) degree are included in the registry.

We analyzed the data of all medical graduates, new ORs, and BCOs in Israel, including age, gender, ethnicity, and country of graduation (i.e., Israeli Medical Graduates (IsrMGs) versus International Medical Graduates (IMGs)) between 2006 and 2021. An international medical graduate (IMG) is a physician who has graduated from a medical school outside of the country where he or she intends to practice. In Israel, similar to other Western countries, the physician workforce is highly diverse, comprising individuals from a wide range of countries (Supplementary Table 1). We included all BCOs up to the age of 78 years, encompassing the age range of practicing ophthalmologists in Israel. Data were directly retrieved in an automated manner from the database in order to minimize human errors. Information on population sizes was obtained from the database of the Israel Central Bureau of Statistics [15].

### Statistical analysis

Data analysis was performed using Microsoft excel (Redmond, Washington, USA) and R software, version 13.1.0 (R Foundation for Statistical Computing, Vienna, Austria). Chi-square tests were used to assess differences in gender and ethnic group distributions among medical graduates, new ORs, and consulting ophthalmologists. Binomial tests were employed to examine annual comparisons between consecutive years. Poisson linear regressions were employed to assess associations between the number of new ORs and BCOs per 100 K, between 2006 and 2021. Both general averages and average yearly change were calculated to assess overall trends as well as central tendencies and consistency. These analyses were then adjusted for medical graduate pool and overall population sizes. Sensitivity analysis was then performed by rerunning the regression analyses without the outlier pandemic year (2020). We found similar results except where specifically noted otherwise. A two-tailed *p*-value < 0.05 was considered statistically significant.

## Results

### Medical graduates

Between 2006 and 2021, a total of 18,624 (41.7% IsrMGs, 42.2% female) medical licenses were issued, with a gradual increase over the years. The number of new licenses corrected to the population size increased from 8.2 to 20.8 per 100 K during 2006–2021, marking a 153% increase. The average yearly increase (AYI) was 6.9% (*p* < 0.001, 95% CI 6.2–7.5%). However, the percentage of new medical licenses granted to IsrMGs and females decreased in 2006–2021 (IsrMGs: 51.4–39.6%; females 49.5–40%) with yearly average declines of 1% and 0.53%, respectively (IsrMGs: *p* < 0.001; 95% CI 0.6–1.4%; females: *p* < 0.001, 95% CI 0.35–0.71%).

### New ophthalmology residents (ORs)

A total of 561 physicians began their ophthalmology residency between 2006 and 2021 (69.9% IsrMGs, 42.5% female, 75% Jewish, average age 31.2 years [male: 31.3; female: 31.1]), with an average yearly percent of females and IsrMGs of 41.0% and 67.4%, respectively. The mean duration between graduation and starting residency was 13.7 months, with a median of 5.5 months.

**Fig. 1:**
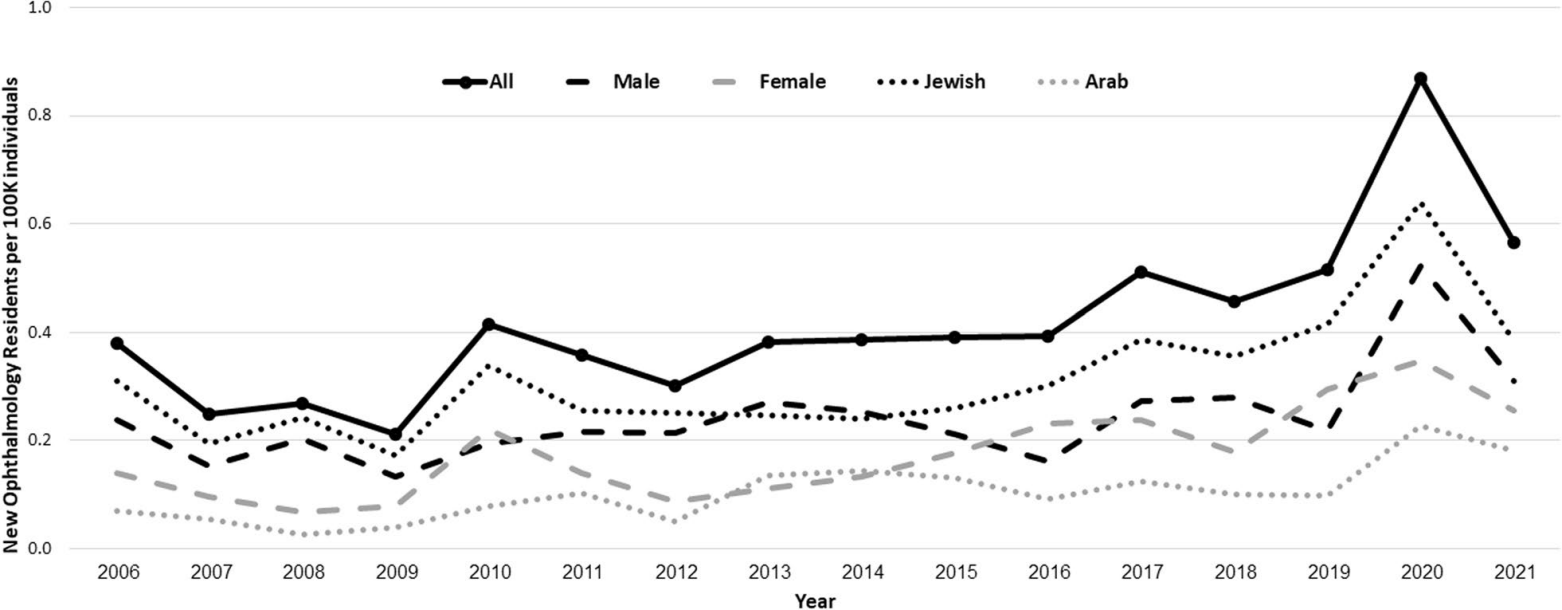
New ophthalmology residents per 100 K individuals during 2006–2021

The number of new ORs increased from 0.38 to 0.57 per 100 K (density) during 2006–2021 (Table 1). Poisson regression revealed a significant 6.2% yearly increase (*p* < 0.001, 95% CI 3.2–9.2%) (Fig. 1). The density of female residents increased from 0.14 to 0.26 and for males from 0.24 to 0.31 (82.2% vs. 29.5%, respectively, *p* < 0.002) (Table 1). The mean proportion of female resident over the years (41%) was notable, yet not significantly different from, their proportion in the general population (50.4%) (*p* = 0.37). Poisson regression revealed significant average annual increases in new female and male residents per 100 K individuals during the study period (female: 8.1%, *p* < 0.001, 95% CI 4.8–11.4%; male: 4.8%, *p* = 0.007, 95% CI 1.3–8.4%).

**Table 1:** Gender distribution, densities, and percent change among new residents during 2006–2021

		All residents			Female residents				Male residents		
Year	Total Population (Per 100 K)	N	Density	% Change from previous year (%)	N	Percent of all	Density	% Change from previous year (%)	N	Density	% Change from previous year (%)
2006	71.16	27	0.38		10	37.0%	0.14		17	0.24	
2007	72.43	18	0.25	− 34.50	7	38.9	0.10	− 31.23	11	0.15	− 36.43
2008	74.19	20	0.27	8.48	5	25.0	0.07	− 30.26	15	0.20	33.14
2009	75.52	16	0.21	− 21.41	6	37.5	0.08	17.89	10	0.13	− 34.51
2010	76.95	32	0.42	96.28	17	53.1	0.22	178.07	15	0.19	47.21
2011	78.36	28	0.36	− 14.08	11	39.3	0.14	− 36.46	17	0.22	11.29
2012	79.84	24	0.30	− 15.87	7	29.1	0.09	− 37.54	17	0.21	− 1.85
2013	81.34	31	0.38	26.78	9	29.0	0.11	26.20	22	0.27	27.03
2014	82.96	32	0.39	1.21	11	34.3	0.13	19.83	21	0.25	− 6.41
2015	84.63	33	0.39	1.10	15	45.4	0.18	33.68	18	0.21	− 15.97
2016	86.28	34	0.39	1.06	20	58.8	0.23	30.78	14	0.16	− 23.71
2017	87.97	45	0.51	29.81	21	46.6	0.24	2.98	24	0.27	68.13
2018	89.67	41	0.46	− 10.61	16	39.0	0.18	− 25.25	25	0.28	2.20
2019	91.40	47	0.51	12.47	27	57.4	0.30	65.56	20	0.22	− 21.51
2020	92.15	80	0.87	68.83	32	40.0	0.35	17.56	48	0.52	138.05
2021	93.71	53	0.57	− 34.85	24	45.2	0.26	− 26.25	29	0.31	− 40.59

Poisson model using gender and year as main factors and their interactions revealed both gender and year to be significant (*p* = 0.003, *p* < 0.001, respectively), with no significant interactions (*p* = 0.18). During 2006–2021, the number of new female residents was 26.3% lower as compared to male residents (ratio = 0.263, *p* = 0.003, 95% CI 0.100–0.396). This density difference remained significant after correcting for their respective populations and medical graduate proportions (*p* = 0.002 and *p* = 0.002, respectively).

Interestingly, in sensitivity analysis, when the outlier year of 2020 was excluded, there were no significant differences in the density of male and female residents as of 2015 (*p* = 0.52).

During 2006–2021, the density of new Jewish ORs increased from 0.3 to 0.38, whereas for Arabs it increased from 0.07 to 0.18 (24.3% and 158.2%, respectively, *p* < 0.001) (Table 2). During the same period, Arabs comprised 20.6% of the population (19.8% in 2006; 21.1% in 2021) and 24.2% of new ORs (18.5% in 2006; 32.1% in 2021). There was no significant difference in the proportion of Arab residents after correcting to their population proportion (21%, *p* = 0.32).

**Table 2:** Ethnicity distribution, densities, and percent change among new residents during 2006–2021

		All residents			Arab residents				Jewish residents		
Year	Total Population (Per 100 K)	N	Density	% Change from previous year (%)	N	Percent of all (%)	Density	% Change from previous year (%)	N	Density	% Change from previous year (%)
2006	71.16	27	0.38		5	18.5	0.07		22	0.31	
2007	72.43	18	0.25	− 34.50	4	22.2	0.06	− 21.40	14	0.19	− 37.48
2008	74.19	20	0.27	8.48	2	10.0	0.03	− 51.18	18	0.24	25.53
2009	75.52	16	0.21	− 21.41	3	18.7	0.04	47.36	13	0.17	− 29.05
2010	76.95	32	0.42	96.28	6	18.7	0.08	96.28	26	0.34	96.28
2011	78.36	28	0.36	− 14.08	8	28.5	0.10	30.93	20	0.26	− 24.47
2012	79.84	24	0.30	− 15.87	4	16.6	0.05	− 50.93	20	0.25	− 1.85
2013	81.34	31	0.38	26.78	11	35.4	0.14	169.93	20	0.24	− 1.84
2014	82.96	32	0.39	1.21	12	37.5	0.14	6.96	20	0.24	− 1.96
2015	84.63	33	0.39	1.10	11	33.3	0.13	− 10.14	22	0.26	7.84
2016	86.28	34	0.39	1.06	8	23.5	0.09	− 28.66	26	0.30	15.92
2017	87.97	45	0.51	29.81	11	24.4	0.13	34.85	34	0.39	28.25
2018	89.67	41	0.46	− 10.61	9	21.9	0.10	− 19.73	32	0.36	− 7.66
2019	91.40	47	0.51	12.47	9	19.1	0.10	− 1.89	38	0.42	16.50
2020	92.15	80	0.87	68.83	21	26.2	0.23	131.44	59	0.64	54.00
2021	93.71	53	0.57	− 34.85	17	32.0	0.18	− 20.40	36	0.38	− 40.00

Poisson regression analysis of residents by ethnic group revealed a significant increase in the density of new Arab and Jewish ORs between 2006 and 2021, with AYIs of 6.4% (*p* < 0.0001, 95% CI 3.3–9.6%) and 5.7% (*p* = 0.0007, 95% CI 2.4–9.1%), respectively.

Similarly, there was a significant effect of ethnicity (*p* < 0.0001), with no year*ethnicity interaction (*p* = 0.21), indicating a lack of trend. The density of Arab ORs was one-third of their Jewish counterparts (ratio: 0.336; 95% CI 0.274–0.412). Correcting for the number of new graduates did not alter the results (ethnicity: *p* < 0.001; year: *p* = 0.99). However, when corrected for population proportions, the difference in density between ethnicities was no longer statistically significant (*p* = 0.097).

Poisson model using type of medical school (IsrMG/IMG) and year as factors and their interaction revealed both medical school origin and year to be significant (*p* = 0.01, *p* < 0.001, respectively), with an interaction between them (*p* = 0.01). Post hoc testing revealed a yearly increase in the density of 7.9% for IMGs (*p* < 0.001), while IsrMGs demonstrated no significant increase (2.2%, *p* = 0.06). Additionally, the yearly average proportion of IMGs was significantly lower than expected based on the graduate pool (32.6% versus 43.5%, *p* < 0.001).

### Board-certified ophthalmologists

**Fig. 2:**
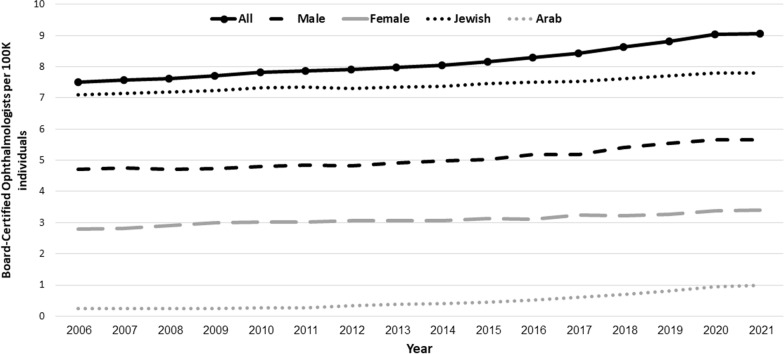
Board certified ophthalmologists per 100 K individuals during 2006–2021

The number of listed BCOs, 78 years of age or younger, ranged from 535 (2006) to 849 (2021) (62.2% IsrMGs, 37.9% female, 10.5% Arab, mean age 54.2 years). Their mean age increased from 51.5 (2006) to 55.1 years (2021). The proportions of female and Arab BCOs were significantly lower than expected based on their respective population proportions (female: 38% vs.50.4%, *p* < 0.001; Arab: 10.5% vs.20.7%, *p* < 0.001). In addition, the yearly average proportion of IMGs was also significantly lower than expected based on the graduate pool (37.6% versus 43.6%, *p* < 0.001). IsrMG and IMG AYIs were 0.15% vs. 0.08% (*p* < 0.001 and *p* = 0.013), respectively. BCO densities were 7.5 in 2006 and 9.06 in 2021, a 20.5% increase. Poisson regression showed a significant AYI of 1.3% during 2006–2021 (*p* < 0.001, 95% CI 0.99–3.2%) (Fig. 2).

During 2006–2021, the density of female BCOs increased from 2.8 to 3.4 while for males it increased from 4.72 to 5.67 (21.3% vs. 20% increase, respectively) (Table 3). Poisson regression analysis of female and male BCO densities revealed significant AYIs (females: 1.38%, *p* < 0.001, 95% CI 1.12–1.61%; males: 1.15%, *p* < 0.001, 95% CI 1.02–1.29%). A Poisson model with gender and year as factors and interaction between them revealed no interaction gender*year interaction (*p* = 0.61) but there was a significant difference for gender (*p* < 0.001) and female BCO density was nearly half that of males (*p* < 0.001). This remained significant even after correcting for their respective population proportions (*p* < 0.001).

**Table 3:** Gender distribution, densities, and percent change among board-certified ophthalmologists during 2006–2021

		All Ophthalmologists			Board-Certified Female Ophthalmologists				Board-Certified Male Ophthalmologists		
Year	Total Population (Per 100 K)	N	Density	% Change from previous year (%)	N	Percent of all (%)	Density	% Change from previous year (%)	N	Density	% Change from previous year (%)
2006	71.16	535	7.52		199	37.2%	2.80		336	4.72	
2007	72.43	549	7.58	0.82	204	37.2	2.82	0.72	345	4.76	0.88
2008	74.19	566	7.63	0.66	216	38.2	2.91	3.38	350	4.72	− 0.95
2009	75.52	583	7.72	1.19	226	38.8	2.99	2.79	357	4.73	0.20
2010	76.95	602	7.82	1.34	233	38.7	3.03	1.18	369	4.80	1.44
2011	78.36	617	7.87	0.64	237	38.4	3.02	− 0.12	380	4.85	1.12
2012	79.84	631	7.90	0.38	245	38.8	3.07	1.46	386	4.83	− 0.30
2013	81.34	649	7.98	0.96	249	38.4	3.06	− 0.24	400	4.92	1.72
2014	82.96	667	8.04	0.76	254	38.1	3.06	0.01	413	4.98	1.23
2015	84.63	691	8.16	1.56	265	38.4	3.13	2.28	426	5.03	1.12
2016	86.28	716	8.30	1.63	269	37.6	3.12	− 0.43	447	5.18	2.92
2017	87.97	742	8.43	1.64	285	38.4	3.24	3.91	457	5.19	0.27
2018	89.67	774	8.63	2.34	288	37.2	3.21	− 0.86	486	5.42	4.33
2019	91.40	806	8.82	2.16	299	37.1	3.27	1.86	507	5.55	2.35
2020	92.15	833	9.04	2.51	311	37.3	3.37	3.17	522	5.66	2.12
2021	93.71	849	9.06	0.22	318	37.5	3.39	0.55	531	5.67	0.03

From 2006 to 2021, the density of Jewish BCOs increased from 7.11 to 7.79, while for Arabs it increased from 0.25 to 0.98 (9.6% vs. 288% increase, respectively). Poisson regression analysis of Jewish and Arab BCO density revealed significant AYIs (Jewish: 0.61%, *p* < 0.001, 95% CI 0.56–0.671; Arab: 12%, *p* < 0.001, 95% CI 10.64–13.40) (Table 4).

**Table 4:** Ethnicity distribution, densities, and percent change among board-certified ophthalmologists during 2006–2021

		All Ophthalmologists			Board-Certified Arab Ophthalmologists				Board-Certified Jewish Ophthalmologists		
Year	Total Population (Per 100 K)	N	Density	% Change from previous year (%)	N	Percent of all (%)	Density	% Change from previous year (%)	N	Density	% Change from previous year (%)
2006	71.16	535	7.52		18	3.36	0.25		506	7.11	
2007	72.43	549	7.58	0.82	17	3.10	0.24	− 7.21	518	7.15	0.58
2008	74.19	566	7.63	0.66	18	3.18	0.24	3.38	533	7.18	0.46
2009	75.52	583	7.72	1.19	19	3.26	0.25	3.70	546	7.23	0.64
2010	76.95	602	7.82	1.34	20	3.32	0.26	3.31	564	7.33	1.38
2011	78.36	617	7.87	0.64	21	3.40	0.27	3.10	576	7.35	0.28
2012	79.84	631	7.90	0.38	27	4.28	0.34	26.19	584	7.31	− 0.49
2013	81.34	649	7.98	0.96	30	4.62	0.37	9.06	598	7.35	0.51
2014	82.96	667	8.04	0.76	33	4.95	0.40	7.85	612	7.38	0.34
2015	84.63	691	8.16	1.56	37	5.35	0.44	9.91	631	7.46	1.08
2016	86.28	716	8.30	1.63	44	6.15	0.51	16.64	648	7.51	0.73
2017	87.97	742	8.43	1.64	54	7.28	0.61	20.36	662	7.52	0.19
2018	89.67	774	8.63	2.34	63	8.14	0.70	14.46	684	7.63	1.37
2019	91.40	806	8.82	2.16	74	9.18	0.81	15.24	705	7.71	1.12
2020	92.15	833	9.04	2.51	87	10.44	0.94	16.61	719	7.80	1.16
2021	93.71	849	9.06	0.22	92	10.84	0.98	3.98	730	7.79	− 0.16

A Poisson model with ethnicity and year as factors and their interaction revealed ethnicity as significant (*p* < 0.001) and a significant ethnicity*year interaction (*p* < 0.001). Yearly change in BCO density for Arabs was 18.98 times that of their Jewish counterparts corrected population proportions (*p* < 0.001, 95% CI 15.42–20.32). Over the study period, the Jewish BCO density was 6.66 times that of Arab BCO density (*p* < 0.001, rate: 6.66; 95% CI 5.28–8.40). Correction for their respective population proportions did not change the results with a significant effect of ethnicity (*p* < 0.001) and a significant ethnicity*year interaction (*p* < 0.001).

Poisson regression using type of medical school (IsrMG/IMG) and year as factors and their interaction revealed medical school type and year to be significant (*p* = 0.01, *p* < 0.001, respectively) but no interaction was found between type and year (*p* = 0.11). IsrMG BCO density was 1.66 times that of IMG BCO density (1.66; 95% CI 1.64–1.69).

## Discussion

In 2019, a historic milestone was reached in the field of medicine, with women making up the majority of medical students in the USA, according to the Association of American Medical Colleges (AAMC) [16]. Despite these evolving changes, achieving racial and ethnical diversity remains an aspired yet still unaccomplished objective [17].

In Israel, there has been a notable increase in the number of medical licenses each year, rising from 8.2 to 20.8 per 100 K individuals between 2006 and 2021 (42% and 41.7% for females and IsrMGs, respectively). In contrast to the positive trends observed in the US, women in Israel continue to be inadequately represented within the medical graduate population, with a regrettable annual decline of 1%. Similarly, IsrMGs showed a yearly decline of 0.53%. This decline in the proportion of IsrMGs is mainly explained by an increase in proportion of IMGs (50.6% IsrMG vs. 49.4 IMG in 2005; 39.6% vs. 60.4% in 2021). The slowly growing allocated spots in Israeli medical schools cannot compete with the vast opportunities for Israeli citizens to study abroad in various international medical schools. This is also likely the cause for the decrease in women representation of all new licenses, as they are underrepresented in the IMG pool (e.g., 54% of IsrMGs vs merely 28% of IMGs in 2021). This serves as an explanation to the rise in the representation of Arabs who consisted 33% of all IMGs in 2006, and 78% in 2021 (nearly 60% of all IMGs that year were Arab men).

These trends, alongside the substantial share of IMGs in the Israeli healthcare workforce is particularly concerning, due to the newly introduced “Yatziv” reform that would revoke many graduates of international schools that have been found inadequate from obtaining a medical license in Israel. This in turn could contribute to an already existing shortage of physicians, and emphasizes the need for additional local, accredited medical schools. As a result, it would likely affect the workforce in many fields. It should be noted, however, that ophthalmology is not likely to be significantly affected due to the competitiveness of the field and the relative ease at which residency spots are filled [18, 19].

A total of 561 physicians started their ophthalmology residency in Israel during 2006–2021. Of these, 75% were Jewish, 69.9% IsrMGs, and 57.5% male. During that time, their density increased from 0.38 to 0.57 per 100 individuals, corresponding to a 6.2% yearly increase. This rate of increase is currently sufficient with the growing density of BCOs and is of tremendous importance in addressing the impending shortage of ophthalmologists that many countries are facing globally, especially with the growing aging population [20, 21].

A previous study has shown that both women and some minorities are underrepresented in the USA among residents in ophthalmology, despite an available pool of eligible medical students [5]. In our cohort, female resident density showed a notably swifter increase (0.14–0.26), as opposed to their male counterparts (0.24–0.31), signifying an optimistic 85.7% vs. 29.2% increase, respectively, over the study duration, with AYIs of 8.1% vs. 4.8%, respectively, but have not yet closed the gender gap during the studied period. Female physicians were found to be slightly, albeit not significantly, underrepresented relative to their proportion in the population. This discrepancy can be attributed to the small sample sizes; however, the representation of female physicians did align with their representation in the medical graduate pool. These trends suggests a positive change in future representation of female physicians in ophthalmology, as opposed to the findings of a recent study in the USA [2]. Of note, excluding the outlier year of 2020, affected by the Covid-19 pandemic, as of 2015 there were no gender disparities among new Israeli ORs. Considering the significant predominance of male residents in the early years of this study, this indicates a substantial increase in the proportion of female residents over the years. These disparities worldwide were previously explained by the lack of sufficient women representation in faculty; however, this does not seem to be the case in Ophthalmology in Israel [2, 13].

Arabs constituted 30% of new ORs, and their density increased significantly over the years (0.07–0.18). Furthermore, their density grew at a substantially faster rate as compared to that of Jewish ORs (0.3–0.38; percentage growth 158.2% versus 24.3%, respectively). This translated into average yearly increments of 6.4% for Arabs and 5.7% for Jewish ORs, reflecting a substantial growth in representation in both groups. However, in contrast to the observed gender differences, no ethnic differences were evident after adjusting for the respective population proportions, aligning the percentage of ORs with the general Israeli population. The presence of gender disparity and lack of an ethnic one may be attributed to various factors including prejudice, societal expectations, or even personal choices as seen in previous studies [2, 22, 23]. This can be explained by the findings of a recent study on Israeli medical students, which revealed that male students displayed a stronger preference for surgical specialties. They were also more willing to delay the initiation of their residencies and more open to starting residencies in specialties that were not their top preferences [24]. Other factors may come into place, such as men working longer hours and having more published research, or women experiencing more harassment in the workplace. This alongside aforementioned personal priorities—affect later career decisions including fellowships, with male predominance in cornea/cataract/refractive and female predominance in pediatric ophthalmology & strabismus and neuro-ophthalmology [13].

There were 7.5 and 9.06 Israeli BCOs per 100 K in 2006 and 2021, respectively, marking a 20.5% rise, with an AYI of 1.3%. This positive trend maintains Israel in a satisfactory position worldwide, and even compared to other western country. For comparison, the density is lower in the US and UK (5.68 and 4.4 UK, respectively), similar in France and Germany, and higher European countries (10 in Belgium and the Czech Republic, 12 in Italy and Spain, and 14 in Greece) [20, 25, 26].

The total breakdown was 62.2% IsrMG, 37.9% female and 10.5% Arab. The proportions of female, Arab, and IMG BCOs were significantly lower than expected based on their respective populations and graduate pool proportions. Female BCO density increased somewhat more than male BCO (2.8 to 3.4, 21.3% and 4.72 to 5.67, 20%, respectively) with AYIs of 1.38% and 1.15%, respectively, and a constant rise in the proportion of BCO females in contrast to USA data [2]. In line with our findings, the Royal College of Ophthalmologists reported that in 2022, although only 37% of BCOs in the UK were female, this represented an increase from the 31% proportion seen in 2018 [27].

The fluctuation in BCO ethnicities was far more pronounced than the trends in the gender gap. Jewish BCO density increased by only 9.6% (7.11–7.79) as opposed to a striking 288% increase for Arab BCOs (0.25–0.98), with respective 0.61% and 12% AYIs. The initial disparity was significant to the extent that even after accounting for population proportions, there was a considerably higher number of Jewish BCOs than expected. These disparities of sub-populations underrepresented in medicine (URM) are often attributed to cultural or socioeconomic differences [28]. In the US, reporting of Hispanic students not envisioning themselves attending higher education, and Black students reporting negative social pressures for educational achievements [29, 30]. In Israel, a study reported Arab women to be more pressured into taking traditional gender-roles, which affect future educational and career opportunities [31]. Additionally, while the Arab population in Israel faces socioeconomic challenges, including lower income and education levels, over half of IsrMGs are in the top economic decile, with a staggering 80% in the top three [32, 33]. Nevertheless, the current trend of increasing diversity is expected to progressively reduce the ethnicity gap in the foreseeable future.

Ophthalmology is considered a competitive specialty. In the USA, IMGs constitute a significant 25% of the medical workforce yet a mere 5% when considering the matching rate for new ORs [34]. A similar, although less pronounced, effect exists in Israel as well, with the majority of both new ORs and BCOs being IsrMGs, even though the majority of the medical workforce in Israel consists of IMGs.

In 2001–2008, in an attempt to diversify the student body in Israel, four Israeli “first-tier” universities implemented a voluntary affirmative action policy for applicants from disadvantaged backgrounds [14]. This, in contrast to Diversity, Equity and Inclusion (DEI) policies in the US, was race-, ethnicity- and individual financial status-neutral. The emphasis was, however, on neighborhood socioeconomic status and high school rigor [14]. These policies, taking into account the seven-year duration of medical education in Israel, roughly corresponds with the year (2015) in which racial and ethnic gaps were bridged. Albeit not applying them to race nor using further measures in residency applications, this implies that in the case of Israel, this policy may have been sufficient to achieve equity even in later career stages, in one of the most competitive fields in medicine, ophthalmology. Another likely contributor to the racial-ethnic gap being resolved is simply the increasing proportion of IMGs among newly licensed physicians in Israel in recent years, as they were predominantly Arab men. Therefore, the ‘Yatziv’ reform could directly impact their representation in the upcoming years. Although the shortage of physicians is currently being addressed through efforts to open new medical schools in Israel, we recommend keeping women and minority representation a key focus. This can be mitigated through additional scholarships to low-income populations, cultural and social support for Arab women, striving for representation in faculty and leadership positions in clinical settings and academia, as well as including diversity in healthcare curriculum to improve awareness [18].

This study has several limitations. The first is its retrospective nature, limiting our ability to assess additional factors. Furthermore, due to the lack of a national matching system, we were unable to assess applicants and determine the degree to which personal choices may have contributed to the observed disparities. Lastly, the level of generalizability could not be assessed. However, previous studies from Israel have shown reliable extrapolation capabilities to other western countries [21, 35–38].

## Conclusions

In conclusion, our comprehensive nationwide study spanning 16 years underscores significant gender- and ethnic- disparities among BCOs that cannot be attributed to differences in population distribution. Despite this, the current analysis of the state of OR's shows positive, rapid changes that will likely mitigate these disparities and enhance diversity in the near future. While women are gradually being more appropriately represented, some differences remain, though they are not statistically significant. Ethnic-racial disparities have also been resolved. This may be the cause of race-neutral affirmative action policies introduced in the 2000s, or merely the increasing proportion of IMGs among newly licensed Israeli physicians, many of whom are Arab men. Pursuing this objective is of paramount importance, as diversity in the workforce can improve the quality of patient-care and have a positive impact on financial outcomes [39] Health policy decision-makers should consider the potential implications of the ‘Yatziv’ reform, which may hinder ethnic minority representation.

## Supplementary Information

Supplementary material 1.

## Data Availability

The data utilized in this study was obtained from the Health Workforce and Infrastructure Forecast (HWIF) division within the Israeli Ministry of Health (MOH). As per data sharing policies and ethical considerations, parties interested in accessing the data should direct their requests to the HWIF division at the Israeli Ministry of Health. Alternatively, researchers may contact the authors directly, providing reasonable justification for their request.

## Abbreviations

AYI: Average yearly increase
BCO: Board-certified ophthalmologists
IMGs: International medical graduates
IsrMGs: Israeli medical graduates
ORs: Ophthalmology residents
