# Supplementary material for: Have gender and ethnic disparities in ophthalmology disappeared? Insights from a workforce-based study in Israel (2006–2021)
Source: Isr J Health Policy Res. 2025 Jan 10;14:2. doi: 10.1186/s13584-024-00664-2 (PMC11720563; doi:10.1186/s13584-024-00664-2)
Supplement: Supplementary file 1 — Supplementary material 1. [file 13584_2024_664_MOESM1_ESM.docx]

Supplementary Table 1. Country of Graduation of Israeli Physicians: 2021 Data Breakdown

| Country of graduation | Percentage |
| --- | --- |
| Israel | 39.2% |
| Russia | 19.3% |
| Romania | 6.2% |
| Italy | 5.2% |
| Ukraine | 4.8% |
| Hungary | 3.5% |
| Moldova | 2.1% |
| United States | 1.9% |
| France | 1.8% |
| Argentina | 1.7% |
| Germany | 1.5% |
| Jordan | 1.5% |
| Former USSR | 1.1% |
| Poland | 0.9% |
| United Kingdom | 0.8% |
| Lithuania | 0.8% |
| Egypt | 0.7% |
| South Africa | 0.6% |
| Latvia | 0.5% |
| Syria | 0.5% |
| Judea and Samaria | 0.5% |
| Australia | 0.3% |
| Brazil | 0.3% |
| Canada | 0.2% |
| Spain | 0.2% |
| Bulgaria | 0.2% |
| Czechoslovakia | 0.2% |
| Czech Republic | 0.1% |
| Armenia | 0.1% |
| Other | 3.2% |
